# A reporting and analysis framework for structured evaluation of COVID-19 clinical and imaging data

**DOI:** 10.1038/s41746-021-00439-y

**Published:** 2021-04-12

**Authors:** Gabriel Alexander Salg, Maria-Katharina Ganten, Andreas Michael Bucher, Hannes Goetz Kenngott, Matthias Alexander Fink, Constantin Seibold, Ricarda Elisabeth Fischbach, Kai Schlamp, Carlos Alberto Velandia, Philipp Fervers, Felix Doellinger, Anna Luger, Saif Afat, Uta Merle, Markus K. Diener, Philippe L. Pereira, Tobias Penzkofer, Thorsten Persigehl, Ahmed Othman, Claus Peter Heußel, Matthias Baumhauer, Gerlig Widmann, Konstantinos Stathopoulos, Bernd Hamm, Thomas J. Vogl, Konstantin Nikolaou, Hans-Ulrich Kauczor, Jens Kleesiek

**Affiliations:** 1 Mint Medical GmbH, Heidelberg, Germany; 2 Dept. of Diagnostic and Interventional Radiology, Frankfurt University Hospital, Frankfurt, Germany; 3 Dept. of Diagnostic and Interventional Radiology, Heidelberg University Hospital, Heidelberg, Germany; 4 cv:hci Lab, Institute of Anthropomatics and Robotics, Karlsruhe Institute of Technology, Karlsruhe, Germany; 5 Dept. of Diagnostic and Interventional Radiology with Nuclear Medicine, Thorax Clinic at Heidelberg University Hospital, Heidelberg, Germany; 6 Center of Radiology, Minimally Invasive Therapies and Nuclear Medicine, SLK Hospital Heilbronn GmbH, Heilbronn, Germany; 7 Dept. of Diagnostic and Interventional Radiology, Cologne University Hospital, Cologne, Germany; 8 Dept. of Radiology, Charité Universitätsmedizin Berlin, Berlin, Germany; 9 Dept. of Radiology, Medical University of Innsbruck, Innsbruck, Austria; 10 Dept. of Diagnostic and Interventional Radiology, Tübingen University Hospital, Tübingen, Germany; 11 Dept. of Gastroenterology and Infectious Diseases, Heidelberg University Hospital, Heidelberg, Germany; 12 Dept. of General, Visceral and Transplantation Surgery, Heidelberg University Hospital, Heidelberg, Germany; 13 Berlin Institute of Health, Berlin, Germany; 14 Translational Lung Research Center, Member of the German Center for Lung Research, Heidelberg, Germany; 15 Dept. of Radiology, Institute Jules Bordet, Brussels, Belgium; 16 Computational Radiology, Dept. of Radiology, German Cancer Research Center (DKFZ), Heidelberg, Germany; 17 Translational Image-guided Oncology, Institute for AI in Medicine (IKIM), University Hospital Essen, Essen, Germany

**Keywords:** Medical imaging, Clinical trials, Databases

## Abstract

The COVID-19 pandemic has worldwide individual and socioeconomic consequences. Chest computed tomography has been found to support diagnostics and disease monitoring. A standardized approach to generate, collect, analyze, and share clinical and imaging information in the highest quality possible is urgently needed. We developed systematic, computer-assisted and context-guided electronic data capture on the FDA-approved mint Lesion^TM^ software platform to enable cloud-based data collection and real-time analysis. The acquisition and annotation include radiological findings and radiomics performed directly on primary imaging data together with information from the patient history and clinical data. As proof of concept, anonymized data of 283 patients with either suspected or confirmed SARS-CoV-2 infection from eight European medical centers were aggregated in data analysis dashboards. Aggregated data were compared to key findings of landmark research literature. This concept has been chosen for use in the national COVID-19 response of the radiological departments of all university hospitals in Germany.

## Introduction

Infection with the virus now known as SARS-CoV-2 has developed into an ongoing pandemic, since the first cases in Hubei province, China were reported to the WHO on December 31, 2019. This has led to a global health crisis with substantial impact on both the patients with COVID-19 at the individual level and on socioeconomic systems worldwide.

The high contagiousness of the disease, a substantial proportion of asymptomatic cases and undocumented infections, and dynamic movement patterns have combined to cause rapid dissemination of the infection^1–3^. With cases globally still on the rise and a lack of effective treatments or vaccines, the spread can currently only be slowed down by drastic disease containment efforts^2–8^. The pandemic has shown that existing measures for disease control were inadequate and that even countries with a strong healthcare infrastructure have insufficient critical care resources. The current spread in low-income countries or those with weak healthcare systems is posing challenges.

Along with the more effective containment, the most promising approach to closing the gap in disease management is to broaden the evidence base by expanding research into clinical diagnostics, treatment, and disease monitoring, especially by taking advantage of new technologies and artificial intelligence^9,10^. However, the impact of aggregation and analysis on the individual patient and on cohorts is high. The highest quality standards are therefore mandatory, and can be attained only by using certified medical software that complies with the regulations of the US Food and Drug Administration (FDA) and the European Economic Area (CE marking). The global spread of COVID-19 demands a global response. Hope exists that aggregation of medical research data and knowledge transfer between countries can substantially expedite the understanding and management of COVID-19. However, these efforts are currently hindered by a number of factors. For one, standardized data reporting is often not available, and, in many cases, common standards have not been defined universally and conclusively. Research efforts like the CO-RADS^11^ or COVID-RADS^12^ standard that address these challenges need to be integrated in a workflow system that can be disseminated globally. The need for a global response means that standardized datasets are needed for comparable results. Moreover, essential diagnostic information is generally spread over several information systems for data generation, storage, and reporting. Finally, the definitions of most meaningful diagnostic tests and references are still subject to frequent change, with a high volume of new research findings. This means on the one hand that intensified efforts toward evidence-based clinical diagnosis and treatment along with reliable monitoring are essential. On the other hand, in order to achieve this in a short time, with comparable results and broad scalability, reliable and efficient methods are needed. An efficient electronic data capture (EDC) solution should therefore support to dynamically be updated, reflecting newly discovered relevant imaging findings as they are established in the course of the pandemic. A combination of open access tried and secure software environments, and state-of-the-art artificial intelligence applications might provide suitable solutions^9^. However, these are not currently readily available in the medical imaging domain. Moreover, the wide range of requirements in the generation of optimal evidence-based approaches and randomized controlled trials necessitates a solution capable of aggregating, swiftly and dynamically, the relevant excerpts from diagnostic, clinical, and epidemiological datasets.

Therefore, we wish to initiate an open-access platform for the data collection compliant with commonly developed criteria for data formats and interfaces, using commonly developed criteria for multimodal diagnosis, staging, and monitoring of COVID-19. Therefore, a novel COVID-19 EDC that provides a direct linkage of image and text annotation in a structured open data format for joint international collaboration is developed. The proposed platform represents the mandatory foundation for efficient, fast, and dynamically adaptable conduct of clinical trials as a prerequisite for novel evidence-based diagnostic and therapeutic discoveries. To address these challenges, the radiology departments of all 36 university hospitals in Germany have combined to set up the RACOON (Radiological Cooperative Network) initiative for joint research into COVID-19 based on the software and cloud-based data platform presented here.

## Results

### Proof of concept: retrospective analysis of multicenter template usage

**Fig. 1:**
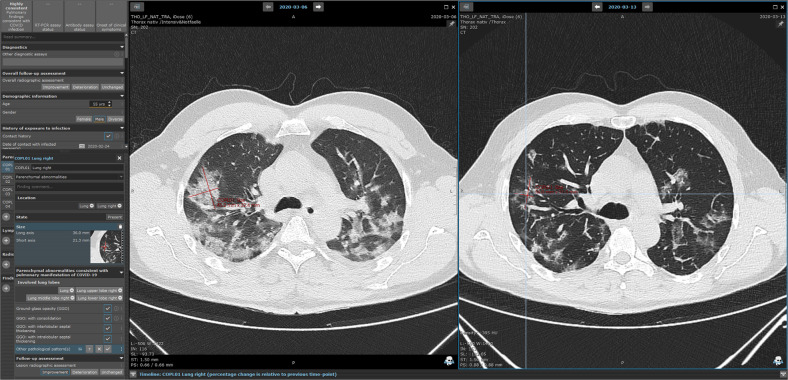
COVID-19 EDC on the mint Lesion^TM^ software platform. In this example, the patient had confirmed COVID-19 and underwent chest CT at two subsequent time points. The images were assessed and evaluated using the Mint EDC by an experienced radiologist. The assessment and evaluation are performed directly on primary imaging data supported by the automatic rule-based evaluation of disease progression. The patient was included in our feasibility clinical trial directly without the necessity for further data collection or redundant documentation.

**Fig. 2:**
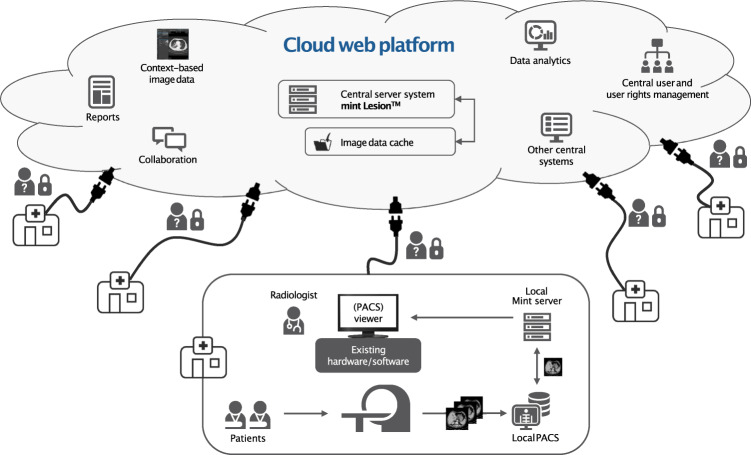
System architecture of a cloud-based web platform. Every healthcare center can upload anonymized data to the web platform and use the Mint EDC based on the mint Lesion^TM^ medical product software package, either by using a local instance or directly via the cloud-based web platform.

In a class 2b/510k certified solution, we developed over a 3-week period a context-sensitive electronic data capture (EDC) template (Fig. 1) that enables systematic large-scale data acquisition to provide an optimized digital platform to gather and aggregate multidisciplinary scientific evidence on COVID-19. Our cloud-based platform includes specific modules for acquisition, storage, analysis, and automated reporting of healthcare data (Fig. 2). The Mint EDC is used for an image-based, both manual and automated data entry process to ensure comparability and uniform data quality, and keep key missing values to a minimum. In order to prove the feasibility of our concept, a retrospective analysis of real-world evidence using the provided tool was performed on data from seven European university hospitals and one community healthcare center. We present the EDC-based data acquisition and data analysis of the exemplary study population, and compare the results to the key findings of three landmark studies.

**Fig. 3:**
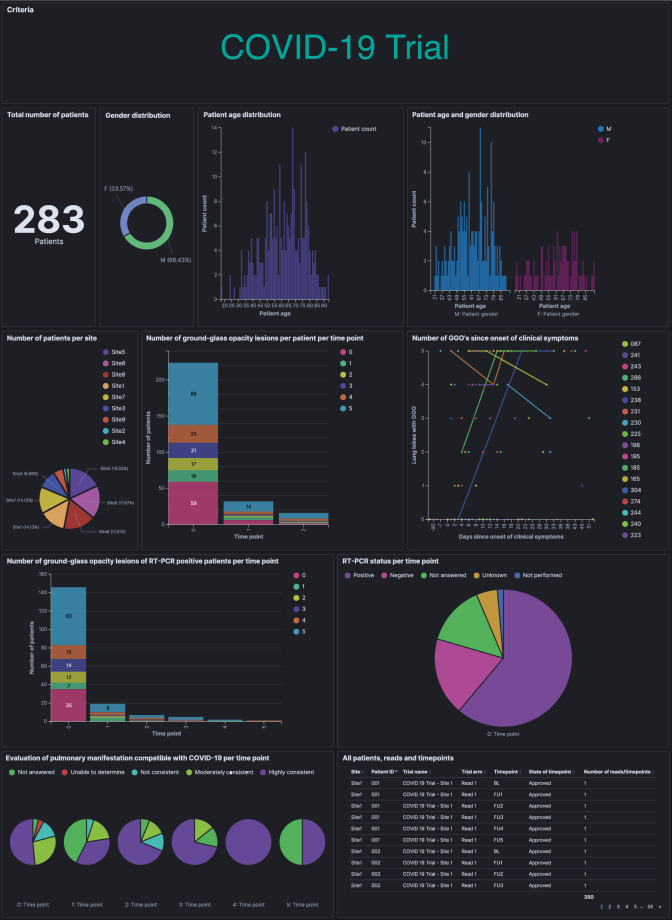
Multicenter data aggregation in a real-time analytics dashboard. Overview of ad hoc COVID-19 usage analysis, including 283 patients (upper left) from eight European hospitals. The data analysis dashboard is made publicly available on https://covid19.mint-imaging.com (for details see “Data availability” section).

The study population comprised 283 inpatients and outpatients from eight European medical centers, who received a clinically indicated chest computed tomography (CT) examination and who were either suspected or known (with laboratory confirmation) to be infected with COVID-19. The selected medical centers provided different levels of care. At each center, radiological assessment, reporting, and data collection were performed independently of this retrospective study. The completeness of the structured reporting items was at the discretion of the examining radiologist. The extracted data were visualized using a real-time statistics dashboard integrated into the software and highly customizable by the user (Fig. 3, for public dashboard access see “Data availability” section). The aim of this usage analysis was to prove the applicability of the concept for coherent multicenter data acquisition by template-based reporting. Therefore, key research literature was consulted to validate the eligibility of this approach. A systematic review and meta-analysis of data pooled from 13 studies provided evidence on the radiological features of pulmonary manifestations of COVID-19 in chest CT^13^. Normalized, pooled data provided in the meta-analysis were used for comparison with the data obtained with our COVID-19 template submitted to real-time data analysis, displaying similar results (Table 1). We found that in 76.0% of the rt-PCR-positive patients, ground-glass opacity (GGO) lesions were detected at baseline imaging. In the majority of the patients, more than one GGO lesion was documented at baseline imaging (five GGO lesions: 38.4%; four GGO lesions: 11.0%; three GGO lesions: 9.3%; and two GGO lesions: 7.5%). The distribution of the parenchymal abnormalities, including GGO lesions was found to be bilateral (overall 87.5%; rt-PCR positive 93.3%; and rt-PCR negative 32.5%), peripheral (85.3; 87.3; and 76.6%), and affecting a minimum of three lobes (58.9; 63.0; and 32.5%).

**Table 1:** Comparison of aggregated data from an ad hoc usage analysis using the COVID-19 template recommendation and proposed software platform with key research findings.

	Bao et al.	Guan et al.	Zhao et al.	COVID-19 template		
	Meta-analysis	Retrospective, multicentric analysis	Retrospective, multicentric analysis	Multicentric data acquisition		
Characteristics						
Study population	*n* = 2738	*n* = 1099	*n* = 101	*n* = 283^a^	*n* = 173	*n* = 52
rt-PCR test positive		Laboratory-confirmed Sars-CoV-2 infection	Laboratory-confirmed Sars-CoV-2 infection	173	173	0
rt-PCR test negative				52	0	52
Mean age		Median 47 (IQR 35.0–58.0)	44.4 ± 12.3	62 ± 15	61 ± 14	64 ± 17
Male (%)	1517 (55.4)	640 (58.2)	45 (44.6)	188 (66.4)	124 (71.7)	27 (51.9)
Female (%)	1221 (44.6)	459 (41.8)	56 (55.4)	95 (33.6)	49 (28.3)	25 (48.1)
CT imaging findings (%)	(95% CI)					
Ground-glass opacity	83.31 (69.43–93.35)	56.4	86.1	73.7	76.0	47.5
Consolidation	43.97 (32.82–55.45)		43.6	18.4	30.1	21.2
Air bronchogram	46.46 (17.16–76.95)			11.0	17.9	11.5
Pleural effusion	5.88 (3.38–8.73)		13.9	17.3	17.3	32.7
Pericardial effusion	4.55 (2.09–7.90)			2.5	2.3	5.8
Lymphadenopathy	3.38 (1.00–6.86)		1.0	7.8	7.5	13.5
Lesion distribution (%)	(95% CI)					
Left				4.4	1.8	12.8
Right				8.1	4.9	21.3
Bilateral	78.2 (65.69–88.19)		82.2	87.5	93.3	66.0
Central	10.81 (0.12–41.50)		1.0	14.7	12.7	23.4
Peripheral	76.95 (57.43–91.50)		87.1	85.3	87.3	76.6
Lobes ≥ 3	70.81 (61.75–79.10)			58.9 (*n* = 224)	63.0 (*n* = 146)	32.5 (*n* = 40)
Clinical symptoms (%)				^b^	^b^	^b^
Fever		43.8/88.7^c^	78.2	70.2 (*n* = 198)	77.5 (*n* = 142)	47.7 (*n* = 44)
Cough		67.8	62.4	71.9 (*n* = 199)	74.6 (*n* = 142)	69.6 (*n* = 46)
Sputum production		33.7		16.1 (*n* = 112)	14.3 (*n* = 84)	25.0 (*n* = 24)
Respiratory distress		18.7	1.0	44.0 (*n* = 200)	47.3 (*n* = 146)	34.1 (*n* = 44)
Nausea		5.0	2.0	9.4 (*n* = 171)	10.6 (*n* = 123)	7.5 (*n* = 40)
Headache		13.6		18.5 (*n* = 173)	21.4 (*n* = 126)	10.3 (*n* = 39)
Diarrhea		3.8	3.0	11.0 (*n* = 172)	11.4 (*n* = 123)	12.2 (*n* = 41)

All COVID-19 EDC data refer to the time of baseline imaging.

^a^Including all patients: rt-PCR positive, negative, not performed and unknown results of PCR test.

^b^Reported data in structured reporting template by radiologist.

^c^On admission/during hospitalization.

Besides the radiological findings, our cohort had clinical presentations similar to those reported in the literature: 77.5% of the rt-PCR-positive patients presented with fever (78.2% (ref. ^14^); on admission 43.8% (ref. ^15^)/during hospitalization 88.7% (ref. ^15^)) and 74.6% with cough (62.4% (ref. ^14^) and 67.8% (ref. ^15^)). There were discrepancies in the reporting of respiratory distress (overall 44.0%; rt-PCR positive 47.3%; and rt-PCR negative 34.1%; compared with 1.0% reported with dyspnea by Zhao et al.^14^ and 18.7% reported with shortness of breath by Guan et al.^15^). The discrepancy between the findings might be attributed to the time point of examination in the course of disease, in which patients that present at a later advanced stage show more severe symptoms. In addition, respiratory distress, dyspnea, or shortness of breath are often insufficiently defined in study protocols and lack objective measures. Therefore, either the inclusion of objective endpoints, such as oxygen saturation rate or exact symptom definitions (here: in question complement) can contribute to standardized data acquisition. At the time of first chest CT examination, 173 patients had a confirmed SARS-CoV-2 infection by rt-PCR and 52 had been tested negative (chest CT prior to rt-PCR test or clinical suspicion of COVID-19 despite rt-PCR result). As part of the radiologist’s report, pulmonary findings were assessed to be highly consistent with pulmonary manifestations of COVID-19 in 146 cases (116 rt-PCR positive and 3 negative), moderately consistent in 78 cases (39 rt-PCR positive and 24 negative), and not consistent in 37 cases (10 rt-PCR positive and 22 negative).

### An approach for unified data collection and analysis using comparable criteria

Our concept focuses on quality, integrity, and traceability. The resulting data cloud provides the basis for the exploration of core outcomes and hypothesis generation for future clinical trials. A digital signature of COVID-19, consisting of imaging biomarkers, patient history, and other clinical follow-up data obtained in a reproducible manner, can be established with this method. The structured data can be universally shared and analyzed, and may be used for future studies on diagnostic support systems and risk stratification by machine learning and artificial intelligence. We demonstrated that a standardized EDC procedure can easily be implemented globally by international dissemination of the COVID-19 EDC for this retrospective analysis. Further, the EDC enables international data contribution via the cloud-based platform. Standardized assessment is possible only if the imaging findings are collected and evaluated at a supra-regional, preferably global level using comparable criteria. The Mint EDC template is available free of charge for use in COVID-19 on the mint Lesion^TM^ medical software product platform (Mint Medical, Heidelberg, Germany; Figs. 1 and 2), and has already been used by university hospitals and healthcare providers in eight countries, including severely affected regions of the USA, Italy, Tyrol in Austria, and the majority of the university hospitals in Germany as a reporting solution in routine workflows, in research-focused projects or a combination of either. The Mint EDC not only provides a structured method for evaluating the pulmonary involvement, but is also essential for quantitative assessment of the progression of the disease, both in clinical trials and in daily clinical routine. Besides providing a tool for clinical research, the Mint EDC is integrated in routine patient assessment. We demonstrate that structured data acquisition for clinical research can be achieved within a structured reporting routine workflow at a comparable time expenditure. In addition, automatic report generation and dynamic adaptation of the Mint EDC to the latest scientific evidence might facilitate this integration.

Furthermore, a cloud-based web platform was provided where hospitals and healthcare centers can access the existing data and upload, assess, report, and document their cases (Figs. 1 and 2). In addition, this data platform enables working remotely and can facilitate second opinions. All centers are encouraged to upload their anonymized data to a central registry. The software features an online dashboard that performs exploratory data analysis in real time to monitor the patient’s progress and provide an alert if applicable. In addition, users can customize the data analysis dashboard. Here, we present a dashboard for exploratory analysis that queries shared multicenter metadata that were acquired using the COVID-19 Mint EDC (Fig. 3, for public dashboard access see “Data availability” section). The provided tool also supports the generation of single-center dashboards or dashboards for a specific trial, which only query the same database. All data records submitted to the cloud may be used to enhance the knowledge of COVID-19 and contribute to improved clinical care of COVID-19 patients in the near future.

### Electronic data capture

**Fig. 4:**
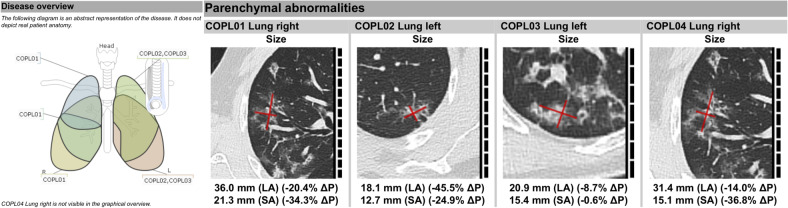
Overview of locations of major findings on chest CT. Longitudinal overview of documented findings on chest CT examination (here: evaluation after second chest CT in COVID-19 disease course). Decrease in the size (LA long axis, SA short axis) of the imaging findings. These findings are included in an automatically generated case report file for a COVID-19-positive patient and study data repository.

A customizable EDC for COVID-19 that covers imaging data and multidisciplinary medical data was developed. The Mint EDC template enables the data acquisition at different levels. In an EDC procedure, recording of document-level patient data, such as demographic information and exposure history, but also data about diagnostic measures already performed and their results, is requested of the user. In addition, secondary clinical data, such as potentially relevant comorbidities, clinical symptoms, and clinical chemistry are queried. The selection of parameters is based on the current study evidence as described previously^16^. A dynamic flow of refinements and amendments is possible with no risk of diminishing the quality of previously collected data. Image assessment includes general pulmonary radiological findings, such as pleural effusion or lymphadenopathy. Pulmonary findings that have been found to be compatible with COVID-19 or in combination with other findings even indicative for COVID-19 are automatically documented in a separate category depending on the ensuing image findings. The EDC template allows for the documentation and measurement of specific findings or general longitudinal monitoring of primary data. Methodological consistency and conformity of the measurements are facilitated by the software architecture, which has been described previously, e.g., for RECIST 1.1 criteria^17^. As an example, once a user documents a parenchymal lesion, he will be asked to specify the location either by choosing from a graphical interface of the lung anatomy or by selection from a prefiltered list of possible locations, for all of which the unique identifier codes from Radiology Lexicon (RadLex), Systemized Nomenclature of Medicine (SNOMED), or NCI Thesaurus are additionally linked. In this EDC, documentation of lesions in the category of parenchymal lung abnormalities, which are not specified to be located within the lung, will lead to an automatic indication of possible irregularities in the conformity of the report. In order to approve the report despite the noncompliance, the user will have to manually justify their decision. Several of these conformity checks have been implemented, in order to guarantee high quality of the report and avoid errors of carelessness, thus ensuring the acquisition of data with comparable quality across sites. Additional automatically computed radiomics features provide secondary data derived from the image that can be used for quantifying the findings. Clinical researchers without programming knowledge can extract radiomics features easily. The measurement of parenchymal abnormalities and the documentation of the grade of the lesion (e.g., ground-glass opacification) at each time point (in the event of several examinations) is annotated to the lobular sublocation in the lung. Bilateral lung involvement, the total number of lung lobes involved, and other qualitative specifications of the lesions with regard to their sublocation are evaluated automatically within a rule-based framework. This leads to an intelligent and simplified workflow supported by logical constraints. A report for each time point and for the longitudinal observation of the patient is generated automatically and can be used for internal communication between departments (see example in Fig. 4; for full report, see Supplementary Notes).

The particular strength of the Mint EDC lies in immediate linkage of imaging-based values not only to the context, e.g., anatomical location, but also to further related data of clinical significance—all in a structured way. Due to the integration in the mint Lesion^TM^ software platform, mineable data are obtained in a semantic data model with a HIPAA- and FDA CFR Part 11-ready audit trail, immediately applicable for phases I–III clinical trials and other medical research. Furthermore, the data are exportable in various human- and machine-readable data formats, such as comma-separated values (.csv) for external analysis. All image annotations can be exchanged in open formats, such as NRRD, or as DICOM-compliant annotations (DICOM RT, DICOM Seg. Surface Obj.). Interfacing with other information systems, such as hospital information systems (HIS) to include clinical data, is possible through standardized interfaces, including Health Level Seven, Fast Healthcare Interoperability Resources (FHIR), and open electronic health records. These interfaces can also be used to receive data and transfer the automated report to the locally used radiological information system, HIS, or other destinations.

## Discussion

The proposed solution supports the detection and standardized recording of clinical and radiological disease features consistent with the course of COVID-19 infection, as well as the relevant epidemiological disease history. Furthermore, a predefined, readily repeatable analysis workflow in the form of a customized analytics dashboard can be used for swift extraction of relevant exploratory statistics. Open access to aggregated anonymized data from this study, usage of the data analysis dashboard, and download of aggregated data is possible via https://covid19.mint-imaging.com, as described in the “Data availability” section. We envisage that this data-driven approach will be used in current and future research into therapies and candidate vaccines for COVID-19, as well as future virus pneumonias, fibrotic diffuse interstitial lung diseases, or other diseases with complex characteristic patterns, as the evaluation of clinical trials on novel therapies and the development of risk stratification markers can be performed using the provided Mint EDC template. In comparison with other global data collection and analysis approaches, such as RICORD (Radiological Society of North America) or the OpenSAFELY platform^18^, the EDC template as one integral part of the presented data collection, and analysis platform will ensure high-quality data, but can also be used for immediate clinical assessment and automatic report generation in clinical routine. In addition, analysis on the provided real-time dashboard can be performed on current, and ongoing data reported via this structured approach due to the systematic and harmonized data collection. As of today, many of the published studies lack a sufficient number of cases or presented only retrospective results with partially incomplete information due to challenges in data acquisition. The ad hoc usage analysis presented herein is itself restricted to an exploratory data analysis due to the relatively small number of cases. Thus, we agree with the authors of other studies that there is a need for larger trials with the systematic data acquisition in the immediate future^15,19^.

We created the Mint EDC template based on current knowledge. As radiologists become more familiar with the radiological findings typical for COVID-19, the proposed EDC will contribute substantially to the diagnosis and treatment of SARS-CoV-2 infection, and facilitate pooling of knowledge across national boundaries. The template, together with its automatically generated reports and computer assistance, might also serve as a learning platform for further education. The rapid spread of COVID-19 in the ongoing pandemic has shown that extensive preparation and adjustment of healthcare systems is crucial, in order to be able to respond appropriately and in timely manner. We hope that the structured guidance through the radiological assessment together with the pooled information gathered from other sites might contribute to flatten the learning curve, and thus improve clinical management of patients suffering from COVID-19. Furthermore, resource scarcity in relation to the number of cases has been widely experienced in terms of medical consumables and infrastructure, but there has also been a shortage of healthcare professionals. A cloud-based web platform obviates the need for physical proximity to the hospitals or protection measures for radiologists. Teleradiology services can readily be enabled based on the proposed concept, thus leading to larger capacities for COVID-19 and other applications.

Our approach has limitations. Data acquisition and handling are more time consuming with an EDC template than a routine clinical reading. Further, integral data acquisition of imaging and clinical parameters requires interdisciplinary input, which might not be available for every case. Template usage statistics have shown that relevant information, e.g., clinical symptoms, that is not directly available to the radiologist may be missed in documentation, resulting in incomplete datasets. Incomplete or missing clinical information is a known issue in radiological assessment that impairs diagnostic accuracy^20^. Thus, the completeness and quality of relevant clinical information are to be considered in radiological reporting^20^. Therefore, “role-dependent” templates can be provided as solutions that integrate into one structured dataset. In the future development of a “role-dependent” EDC, we aim to define task templates that consist of subsets of data items to be automatically assigned to the radiologist, but also to, e.g., other clinicians or study nurses in order to improve workflow efficiency, data quality, and use of human resources. In addition, conformity and interdependence checks, rule-based automatic assistance and automatic data capture, using the FHIR interface might contribute to complete datasets and reduce processing time. Currently, the template is designed for CT imaging only, but an EDC template for chest radiographs could be realized easily and is planned for the future. Further its use could aid in the systematic workup of extrapulmonary imaging findings. Lastly, it might appear illogical to encourage increased research even though manpower is currently limited. The primary goal is to provide a tool for future clinical studies, which are urgently needed.

We do not want to present a predefined global standard for the assessment and reporting of patients with COVID-19; rather, our intention is to initiate an evidence-based approach to define a joint global standard that can be adapted to dynamic situations and to discoveries yet to come. As an example, accumulating evidence indicates a key role of pulmonary vascular pathologies in COVID-19 that can be assessed in chest CT and contribute to improved patient management^12,21–23^. New evidence will be evaluated and integrated in future versions of the Mint EDC. Standardized data acquisition and aggregation may also reveal regional differences in the expression of the disease depending on the respective case load and regional healthcare capacity leading to a dynamic patient management. The Mint EDC can be adapted and refined according to evidence generated in ongoing and future clinical trials, and additional reporting items, e.g., the CO-RADS^11^ or COVID-RADS^12^ assessment, can be added and determined retrospectively. Any addition to or changes in template parameters will not endanger previously collected data. Permanent data integrity is assured, and even retrospective acquisition of certain parameters will be possible if required.

Chest CT as a supporting tool for both diagnosis and treatment monitoring has distinct benefits, such as short turnaround time, wide availability of the necessary equipment, and the possibility of assessing the stage of the disease, including response to treatments. However, it must be emphasized that even though the reporting is conducted directly on the CT image data, the EDC template can be used even if no imaging dataset is available. Parameters regarding the patient history, clinical symptoms, and clinical chemistry could still be documented systematically and shared with other ongoing activities, such as the LEOSS project^24^. Consequently, the target group of users includes not only radiological departments, but every other department that contributes to the diagnosis and treatment of COVID-19 patients.

The presented platform will be used by all 36 German university hospitals in the recently initiated joint nationwide infrastructure initiative RACOON. The cloud-based platform is integral in the development of a unified backbone for data annotation, analysis, and exchange among the partners. With the presented systematic, computer-assisted, and context-guided approach to data capture and annotations based on easy-to-use software, we wish to contribute to a joint global response against the COVID-19 pandemic in a balancing act between research and clinical routine and, moreover, to initiate a change in clinical study culture.

## Methods

### mint Lesion^TM^ software platform

The mint LesionTM platform provides radiologists, physicians, and researchers with a certified medical product software for assessment of radiological images. Users can examine and process these images, identify structures and features, and perform measurements on them. Typical uses are screening, staging, and treatment response monitoring in various types of cancer. User guidance along a detailed workflow process is provided, in which every clinical finding and measurement is tied to the respective metadata. Data collection, annotation, and analysis were performed on a dedicated and private cloud platform hosted in Germany. All data are secured in a geo-redundant backup storage. State-of-the-art encryption for all data in transit and at rest has been implemented by means of the Advanced Encryption Standard-256 technology. Communication security between servers and client web browsers is secured by Transport Layer Security 1.2. Login credentials are personalized and follow a defined password policy. The network storage is subdivided into an image buffer that stores image data as imported via the provided upload/anonymization tool without any changes on the actual images. Annotation, data records etc. are stored in an open-source PostgresSQL database. An independent and renown IT security company has performed an IT security audit prior to the productive use. All observations of the audit have been mitigated during the audit period. The auditors verified that no observations were remaining unresolved.

### Knowledge sources

In March 2020, we published the first version of the EDC using data items based on a scientific literature review and the guidelines of the relevant medical associations^16,25,26^. Due to the highly dynamic development of knowledge about the pathophysiology, clinical presentation, and disease course of COVID-19, continuous adjustments and amendments were necessary for the data items queried in the COVID-19 Mint EDC. Updates of the EDC template are performed based on continuous screening of the scientific literature, and all refinements are validated by medical experts in accordance with the predefined audit trail.

### EDC template development

The template was developed based on the mint Lesion^TM^ software platform allowing for implementation of customized reporting templates. With a total of 67 reporting items, the template consists of document-level data items that provide validity for each patient on a more general level, accompanied by measurement-level data items in the context of a specific annotation on the primary imaging data. Document-level data items included the reporting categories “diagnostics” (rt-PCR test result and antibody assay result), “demographic information”, “history of exposure to infection”, “comorbidities”, “clinical symptoms”, “clinical chemistry”, “general pulmonary radiological findings”, and “COVID-19 compatible radiological findings”. The document-level questions mostly offer a choice of three answers (yes/no/not evaluable), but have been customized where applicable and offer free-text fields where appropriate. On the measurement level, lesions can be documented using a range of annotation tools, including simple maximum diameter or long axis/short axis measurements, but also free-form 2D or 3D segmentation by definition of the region of interest to compute radiomics parameters. Each measurement is documented on the primary image, including its specific anatomical location. Rule-based automatic evaluations and automatic acquisition of data items are implemented where applicable. As an example, the laterality of lung manifestations (left/right/bilateral) and the number of lung lobes involved are automatically derived from the documentation of lesions on the primary imaging data based on their specified location. Conformity checks specific to the COVID-19 reporting template are included to ensure valid documentation of lesions. Wherever applicable, further explanation or literature citations are provided for the queried data items.

### Implementation and dissemination

In March 2020, Mint Medical GmbH, Heidelberg, Germany deployed the COVID-19 Mint EDC at all customer sites per configuration. The COVID-19 EDC template was installed free of charge, and there were no fees for its use. For medical centers that did not already have a local Mint server installed, a cloud-based software platform with secure remote access options was developed and provided free of charge.

### Template-based image assessment

Images were assessed by experienced radiologists at the participating study centers. The radiological departments of the university hospitals of Heidelberg (represented by Dept. of Radiology, Heidelberg University and Dept. of Radiology, Thorax Clinic at Heidelberg University), Tuebingen, Frankfurt, Innsbruck, Cologne, and Berlin, the Institut Jules Bordet in Brussels, and the regional hospital of Heilbronn participated in this study. There was no training in the use of structured reporting templates for patient assessments in general or of the COVID-19 Mint EDC template in particular, apart from the complimentary template description and user manual provided.

### Usage analysis: overview

The analysis was designed by the investigators. Ethical approval for analysis of the de-anonymized metadata was obtained from the appropriate review board by the coordinating study center at Heidelberg University Hospital. Written informed consent was waived owing to the retrospective usage of anonymized metadata. Anonymized data were aggregated and visualized for descriptive analysis.

### Usage analysis: definition and outcome

The primary intention was to prove the feasibility of using the COVID-19 EDC template for patient assessment, and subsequent systematic and standardized data acquisition applicable for epidemiological and clinical research by retrospective analysis of metadata. Therefore, landmark research literature was used for comparison with the results of this unsupervised, ad hoc, investigator-initiated, multicenter metadata analysis.

### Data sources

Usage records of the developed COVID-19 EDC were obtained from all participating study centers. All anonymized metadata were pooled for a central analysis at the provided cloud server. Anonymization was performed in a two-step process via an upload tool. First, all directly identifying attributes were removed consistent with Attribute Confidentiality profile (DICOM PS 3.15: Appendix E), according to DICOM Standard committee (working group 18) Supplement 142. In a second step, the patient ID is manipulated by multiple hashing using a cryptographic method. Confidential information, partially deducted from patient data, partially specific to the user is inserted as salt. The procedure is nonreversible to all participants. However, longitudinal study data will still be assigned correctly. Therefore, at least the level of qualified anonymization as introduced by the Horizon 2020 DS-08-2017 “Cybersecurity PPP: Privacy, Data Protection, Digital Identities” is fulfilled. The inclusion criteria were either laboratory-confirmed or suspected COVID-19 infection and the clinical indication for chest CT in a patient, who was either hospitalized or being treated as an outpatient. Whether continuous cases were selected and whether data were annotated with the EDC was purposely left to the discretion of each participating site.

### Dashboard data aggregation and statistical analysis

The data aggregation and analysis were performed using the software add-on mint Analytics^TM^, which provided the possibility to perform real-time analysis, using a configurable search engine and front end for visualization. The software is open source based on a front-end application (Kibana) for queries and visualization of data indexed in elasticsearch (Elasticsearch, Inc., US, elastic.co). Numerical values were summarized as averages and standard deviations. Categorical variables were expressed as percentages. No imputation was made for missing data. The laterality of the lesions and the number of lobes affected by pathological findings compatible with COVID-19 was derived automatically, using a rule-based framework depending on the number of lesions annotated and their respective locations within the lung. The cohort of patients assessed with this COVID-19 EDC has not been randomized, and decisions on the applicability of EDC template assessment and subsequent inclusion in this analysis were at the discretion of the responsible medical centers; thus, statistics are descriptive only. Baseline imaging time points were chosen for analysis. Subgroup analyses were performed depending on the rt-PCR status reported for each patient and divided into rt-PCR positive, rt-PCR negative, and patients with either unknown rt-PCR status or no test performed.

### Reporting summary

Further information on research design is available in the Nature Research Reporting Summary linked to this article.

## Supplementary information

Reporting Summary

Supplementary Information

## Data Availability

The COVID-19 Mint EDC is demonstrated at http://cloud1.mint-medical.de/downloads/player/index.html?v=Covid19StandardizedAssessmentWeb. Raw data generated with the COVID-19 EDC used for this multicenter analysis remain the property of the respective participating institution. The anonymized datasets of this multicenter usage analysis were aggregated solely for the proof of concept of the proposed data generation and analysis platform. We provide public access to the data analysis dashboard and aggregated anonymized data on https://covid19.mint-imaging.com (User-ID: mint, password: FightCovid!). Every reader has the opportunity to use the dashboard and visualizations, filter for specific data, and download the respective data. The presented data form part of several other medical research projects and investigator-initiated trials and will be included in individual and joint publications. For the German RACOON project, a committee for data access and collaboration will be formed and can be reached via the corresponding author.

## Code availability

No nonproprietary, customized code was used for data analysis. The data analysis dashboard and its framework can be accessed through https://covid19.mint-imaging.com (User-ID: mint, password: FightCovid!).
