# Supplementary material for: A reporting and analysis framework for structured evaluation of COVID-19 clinical and imaging data
Source: NPJ Digit Med. 2021 Apr 12;4:69. doi: 10.1038/s41746-021-00439-y (PMC8041811; doi:10.1038/s41746-021-00439-y)
Supplement: Supplementary file 1 — Reporting Summary [file 41746_2021_439_MOESM1_ESM.pdf]

nature research

Corresponding author(s): Prof. Dr. Dr. Jens Kleesiek

Last updated by author(s): Jan 19, 2021

Reporting Summary

Nature Research wishes to improve the reproducibility of the work that we publish. This form provides structure for consistency and transparency in reporting. For further information on Nature Research policies, see our Editorial Policies and the Editorial Policy Checklist.

Statistics

For all statistical analyses, confirm that the following items are present in the figure legend, table legend, main text, or Methods section.

n/a	Confirmed	
☐	☒	The exact sample size (n) for each experimental group/condition, given as a discrete number and unit of measurement
☒	☐	A statement on whether measurements were taken from distinct samples or whether the same sample was measured repeatedly
☒	☐	The statistical test(s) used AND whether they are one- or two-sided<br>*Only common tests should be described solely by name; describe more complex techniques in the Methods section.*
☒	☐	A description of all covariates tested
☒	☐	A description of any assumptions or corrections, such as tests of normality and adjustment for multiple comparisons
☐	☒	A full description of the statistical parameters including central tendency (e.g. means) or other basic estimates (e.g. regression coefficient) AND variation (e.g. standard deviation) or associated estimates of uncertainty (e.g. confidence intervals)
☒	☐	For null hypothesis testing, the test statistic (e.g. F, t, r) with confidence intervals, effect sizes, degrees of freedom and P value noted<br>*Give P values as exact values whenever suitable.*
☒	☐	For Bayesian analysis, information on the choice of priors and Markov chain Monte Carlo settings
☒	☐	For hierarchical and complex designs, identification of the appropriate level for tests and full reporting of outcomes
☒	☐	Estimates of effect sizes (e.g. Cohen's d, Pearson's r), indicating how they were calculated

Our web collection on statistics for biologists contains articles on many of the points above.

Software and code

Policy information about availability of computer code

Data collection	mint Lesion and mint Analytics, Mint Medical GmbH, Heidelberg, Germany
Data analysis	mint Lesion and mint Analytics, Mint Medical GmbH, Heidelberg, Germany

For manuscripts utilizing custom algorithms or software that are central to the research but not yet described in published literature, software must be made available to editors and reviewers. We strongly encourage code deposition in a community repository (e.g. GitHub). See the Nature Research guidelines for submitting code & software for further information.

Data

Policy information about availability of data

All manuscripts must include a data availability statement. This statement should provide the following information, where applicable:

- Accession codes, unique identifiers, or web links for publicly available datasets
- A list of figures that have associated raw data
- A description of any restrictions on data availability

The COVID-19 Mint EDC is demonstrated at http://cloud1.mint-medical.de/downloads/player/index.html?v=Covid19StandardizedAssessmentWeb. Raw data generated with the COVID-19 EDC used for this multicenter analysis remain the property of the respective participating institution. The anonymized datasets of this multicenter usage analysis were aggregated solely for the proof of concept of the proposed data generation and analysis platform. We provide public access to the data analysis dashboard and aggregated anonymized data on https://covid19.mint-imaging.com (User-ID: mint, password: FightCovid!). Every reader has the opportunity to use the dashboard and visualizations, filter for specific data and download the respective data. The presented data form part of several other medical research projects and investigator-initiated trials and will be included in individual and joint publications. For the German RACOON project, a committee for data access and collaboration will be formed and can be reached via the corresponding author.

Field-specific reporting

Please select the one below that is the best fit for your research. If you are not sure, read the appropriate sections before making your selection.

☒ Life sciences ☐ Behavioural & social sciences ☐ Ecological, evolutionary & environmental sciences

For a reference copy of the document with all sections, see nature.com/documents/nr-reporting-summary-flat.pdf

Life sciences study design

All studies must disclose on these points even when the disclosure is negative.

Sample size	Retrospective, exploratory, descriptive metadata analysis
Data exclusions	Retrospective, exploratory, descriptive metadata analysis
Replication	Retrospective, exploratory, descriptive metadata analysis. No replication.
Randomization	Retrospective, exploratory, descriptive metadata analysis. No randomization.
Blinding	Retrospective, exploratory, descriptive metadata analysis. All data were anonymized before upload to cloud-based web platform. Dashboard analysis.

Reporting for specific materials, systems and methods

We require information from authors about some types of materials, experimental systems and methods used in many studies. Here, indicate whether each material, system or method listed is relevant to your study. If you are not sure if a list item applies to your research, read the appropriate section before selecting a response.

Materials & experimental systems

n/a	Involved in the study	
☒	☐	Antibodies
☒	☐	Eukaryotic cell lines
☒	☐	Palaeontology and archaeology
☒	☐	Animals and other organisms
☒	☐	Human research participants
☐	☒	Clinical data
☒	☐	Dual use research of concern

Methods

n/a	Involved in the study	
☒	☐	ChIP-seq
☒	☐	Flow cytometry
☒	☐	MRI-based neuroimaging

Clinical data

Policy information about clinical studies

All manuscripts should comply with the ICMJE guidelines for publication of clinical research and a completed CONSORT checklist must be included with all submissions.

Clinical trial registration	Retrospective, exploratory, descriptive metadata analysis
Study protocol	Not applicable due to retrospective, exploratory, descriptive metadata analysis
Data collection	Not applicable due to retrospective, exploratory, descriptive metadata analysis
Outcomes	Proof of feasibility, descriptive metadata analysis by comparison with landmark literature
