# Supplementary material for: A reporting and analysis framework for structured evaluation of COVID-19 clinical and imaging data
Source: NPJ Digit Med. 2021 Apr 12;4:69. doi: 10.1038/s41746-021-00439-y (PMC8041811; doi:10.1038/s41746-021-00439-y)
Supplement: Supplementary file 2 — Supplementary Information [file 41746_2021_439_MOESM2_ESM.pdf]

made with mint Lesion

COVID-19 Trial report - 03/13/2020

Covid-19, Nr1 (M) | 03/25/1965 | ID 978647854 | COVID-19 Trial Case

Overall assessment

Diagnostics

Pulmonary findings consistent with COVID infection: Highly consistent

Disease overview

The following diagram is an abstract representation of the disease. It does not depict real patient anatomy.

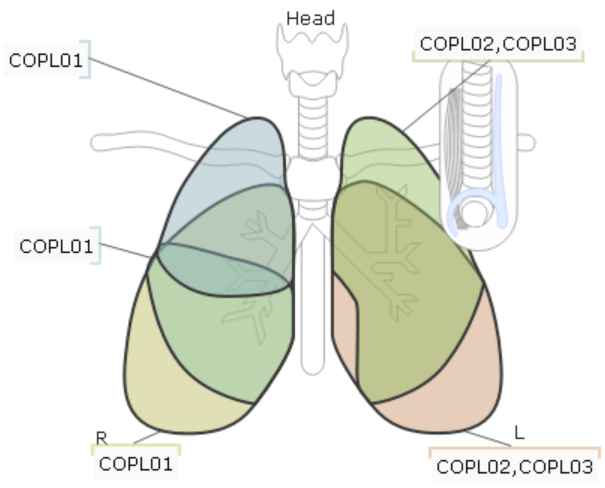

COPL04 Lung right is not visible in the graphical overview.

General

Demographic information

Age: 55 yrs
Gender: Male

History of exposure to infection

Contact history: Present
Date of contact with infected person(s): 02/24/2020
Exposure to high-risk geographic area: Present
Geographic Area: Südtirol

COVID-compatible radiological signs

Distribution pattern: Peripheral
Laterality of lung lobe involvement: Bilateral *(Parenchymal abnormalities defined in both lungs)*
Lung lobes with Ground-glass opacities (GGO): 5 *(GGO present in:, Lung middle lobe right, Lung lower lobe left, Lung upper lobe right, Lung lower lobe right, Lung upper lobe left)*
Lung lobes with GGO with consolidation: 5 *(GGO with consolidation present in:, Lung middle lobe right, Lung lower lobe left, Lung upper lobe right, Lung lower lobe right, Lung upper lobe left)*
Lung lobes with GGO with interlobular septal thickening: 5 *(GGO with interlobular septal thickening present in:, Lung middle lobe right, Lung lower lobe left, Lung upper lobe right, Lung lower lobe right, Lung upper lobe left)*
Lung lobes with GGO with intralobular septal thickening: 5 *(GGO with intralobular septal thickening present in:, Lung middle lobe right, Lung lower lobe left, Lung upper lobe right, Lung lower lobe right, Lung upper lobe left)*

Clinical chemistry

White blood cell count: Increased
Lymphocyte count: Increased
Platelet count: Increased
C-Reactive Protein: Normal

Clinical symptoms

Fever: Present
If fever present, please provide details: 39
Respiratory distress: Present
Headache: Present
Nausea: Not present
Diarrhea: Not present

Comorbidities

Tobacco smoking: No history
Chronic obstructive pulmonary disease: No
Hypertension: No
Cardiac disease: No
Chronic liver disease: No
Diabetes: No

Parenchymal abnormalities

COPL01 Lung right

Size: 36.0 mm (LA) (-20.4% ΔB / -20.4% ΔN / -20.4% ΔP) / 21.3 mm (SA) (-34.3% ΔB / -34.3% ΔN / -34.3% ΔP)
State: Present
Involved lung lobes: Lung upper lobe right, Lung middle lobe right, Lung lower lobe right
Ground-glass opacity (GGO): Present
GGO: with consolidation: Present
GGO: with interlobular septal thickening: Present
GGO: with intralobular septal thickening: Present
Lesion radiographic assessment: Improvement

COPL02 Lung left

Size: 18.1 mm (LA) (-45.5% ΔB / -45.5% ΔN / -45.5% ΔP) / 12.7 mm (SA) (-24.9% ΔB / -24.9% ΔN / -24.9% ΔP)
State: Present
Involved lung lobes: Lung upper lobe left, Lung lower lobe left
Ground-glass opacity (GGO): Present
GGO: with consolidation: Present
GGO: with interlobular septal thickening: Present
GGO: with intralobular septal thickening: Present

COPL03 Lung left

Size: 20.9 mm (LA) (-8.7% ΔB / -8.7% ΔN / -8.7% ΔP) / 15.4 mm (SA) (-0.6% ΔB / -0.6% ΔN / -0.6% ΔP)
State: Present
Involved lung lobes: Lung upper lobe left, Lung lower lobe left

COPL04 Lung right

Size: 31.4 mm (LA) (-14.0% ΔB / -14.0% ΔN / -14.0% ΔP) / 15.1 mm (SA) (-36.8% ΔB / -36.8% ΔN / -36.8% ΔP)
State: Present

Not approved

made with mintLesion

COVID-19 Trial report - 03/13/2020

Covid-19, Nr1 (M)	03/25/1965	ID 978647854	COVID-19 Trial Case

Parenchymal abnormalities

COPL01 Lung right	COPL02 Lung left	COPL03 Lung left	COPL04 Lung right
**Size**	**Size**	**Size**	**Size**
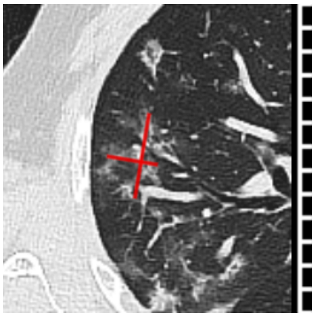	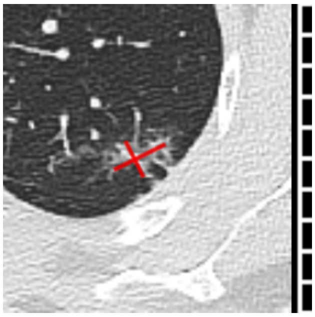	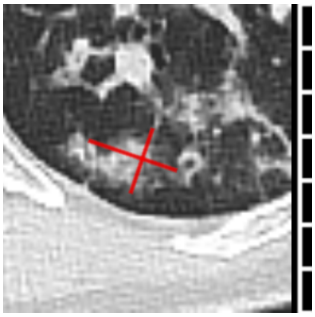	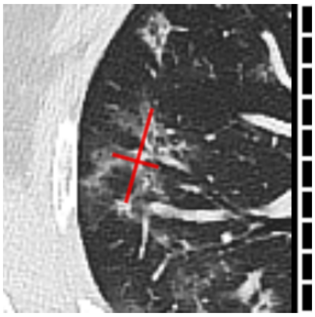
**36.0 mm (LA) (-20.4% ΔP)**<br>**21.3 mm (SA) (-34.3% ΔP)**	**18.1 mm (LA) (-45.5% ΔP)**<br>**12.7 mm (SA) (-24.9% ΔP)**	**20.9 mm (LA) (-8.7% ΔP)**<br>**15.4 mm (SA) (-0.6% ΔP)**	**31.4 mm (LA) (-14.0% ΔP)**<br>**15.1 mm (SA) (-36.8% ΔP)**

Conformity check

General

- Lung lobe not chosen for COPL04. Precise location influences count of lung lobes with COVID-compatible radiological signs.

Not approved
